# Epitope-Based Immunoinformatic Approach on Heat Shock 70 kDa Protein Complex of *Cryptococcus neoformans* var. *grubii*

**DOI:** 10.1155/2021/9921620

**Published:** 2021-08-19

**Authors:** Reham M. Elhassan, Nagla M. Alsony, Khadeejah M. Othman, Duaa T. Izz-Aldin, Tamadour A. Alhaj, Abdelrahman A. Ali, Lena A. Abashir, Omar H. Ahmed, Mohammed A. Hassan

**Affiliations:** ^1^Department of Biotechnology, Africa City of Technology, Khartoum, Sudan; ^2^Department of Pharmaceutical Chemistry, Faculty of Pharmacy, Sudan International University, Khartoum, Sudan; ^3^Department of Microbiology, Faculty of Medical Laboratory Science, Kamlin Ahlia College, Gazira, Sudan; ^4^Department of Microbiology, Faculty of Medical Laboratory Science, Sudan University for Science and Technology, Khartoum, Sudan; ^5^Department of Microbiology, Abu Huzaifa Health Center, Khartoum, Sudan; ^6^Department of Molecular Biology, Institute of Endemic Disease, University of Khartoum, Khartoum, Sudan; ^7^Department of Neurosurgery, Ribat University Hospital, Khartoum, Sudan; ^8^Department of Pharmacy, Fedail Hospital, Khartoum, Sudan; ^9^Department of Pharmacology, Faculty of Pharmacy, University of Gazira, Wad Medany, Sudan; ^10^Department of Bioinformatics, DETAGEN Genetic Diagnostics Center, Kayseri, Turkey

## Abstract

**Introduction:**

Cryptococcosis is a ubiquitous opportunistic fungal disease caused by *Cryptococcus neoformans* var. *grubii*. It has high global morbidity and mortality among HIV patients and non-HIV carriers with 99% and 95%, respectively. Furthermore, the increasing prevalence of undesired toxicity profile of antifungal, multidrug-resistant organisms and the scarcity of FDA-authorized vaccines were the hallmark in the present days. This study was undertaken to design a reliable epitope-based peptide vaccine through targeting highly conserved immunodominant heat shock 70 kDa protein of *Cryptococcus neoformans* var. *grubii* that covers a considerable digit of the world population through implementing a computational vaccinology approach.

**Materials and Methods:**

A total of 38 sequences of *Cryptococcus neoformans var. grubii*'s heat shock 70 kDa protein were retrieved from the NCBI protein database. Different prediction tools were used to analyze the aforementioned protein at the Immune Epitope Database (IEDB) to discriminate the most promising T-cell and B-cell epitopes. The proposed T-cell epitopes were subjected to the population coverage analysis tool to compute the global population's coverage. Finally, the T-cell projected epitopes were ranked based on their binding scores and modes using AutoDock Vina software. *Results and Discussion*. The epitopes (ANYVQASEK, QSEKPKNVNPVI, SEKPKNVNPVI, and EKPKNVNPVI) had shown very strong binding affinity and immunogenic properties to B-cell. (FTQLVAAYL, YVYDTRGKL) and (FFGGKVLNF, FINAQLVDV, and FDYALVQHF) exhibited a very strong binding affinity to MHC-I and MHC-II, respectively, with high population coverage for each, while FYRQGAFEL has shown promising results in terms of its binding profile to MHC-II and MHC-I alleles and good strength of binding when docked with HLA-C∗12:03. In addition, there is massive global population coverage in the three coverage modes. Accordingly, our *in silico* vaccine is expected to be the future epitope-based peptide vaccine against *Cryptococcus neoformans* var. *grubii* that covers a significant figure of the entire world citizens.

## 1. Introduction

Cryptococcosis is a ubiquitous opportunistic infection caused by *Cryptococcus neoformans* that causes life-threatening pneumonia and meningoencephalitis in immunocompromised patients [1–3]. Cryptococcal infection is considered one of the most predominant causes of death globally with an estimated annual mortality of 624,700 cases [4–6]. Commonly, *Cryptococcus neoformans* var. *grubii* strains (serotype A) are more virulent, are widely distributed all over the world, and cause 99% of infections in HIV patients and 95% of the non-HIV carriers [7, 8].

Clinical and experimental evidence suggests that cell-mediated immunity by T helper CD4+ plays a crucial contribution to host defense against intracellular cryptococcosis [9–12]. Nevertheless, this does not contradict the importance of B-cell response, which is considered a vital mechanism for inducing protection against cryptococcosis in individuals with impaired cell-mediated immunity [13–15]. When it comes to immunomodulatory factors, it is given that *C. neoformans* expresses a significant number of immune-proteomic factors that evoke host immunity that could be useful targets as diagnostic markers or vaccines [16–20]. Interestingly, heat shock 70 kDa protein (Hsp70) is one of the novel immunogenic proteins that trigger cellular and humoral response against murine pulmonary cryptococcosis and cryptococcal meningitis [19, 21–23]. Throughout evolution, the Hsp70 family is a highly conserved cell-surface protein and widely expressed in Plasmodium [24], Trypanosoma, Schistosoma, Leishmania [25], Toxoplasma [26], *Candida* [27, 28], *Histoplasma* [29, 30], and *Mycobacterium* [31] species.

Cryptococcosis has become more prevalent and the main cause of morbidity and mortality in the last 30 years for the eruption of the HIV epidemic [32]. Despite advances in therapy, the current antifungal therapies such as azoles, polyenes, and echinocandins are not effective in eradicating the pathogens, and even with treatment, there is high mortality and morbidity; infusion toxicity and renal impairment can take place during amphotericin B treatment [33], and bone marrow suppression can also occur during 5-flucytosine therapy [34]. Moreover, there is a high burden of life-threatening Cryptococcus-related immune reconstitution inflammatory syndrome (IRIS) in HIV individuals [35]. Yet, to date, there is no FDA-authorized vaccine available to combat cryptococcosis. Thus, this underlies an urgent need for designing a reliable immunome-derived epitope-driven vaccine through mapping the parasite's immunoreactive proteins using different computational software [36–38]. In this study, we aimed to design an epitope peptide vaccine of highly conserved immunodominant B and T lymphocyte epitopes with a wide global population coverage design against *Cryptococcus neoformans* var. *grubii* by implementing an emerging approach in computational vaccinology. This strategy is quite advantageous because it speeds up the process of successful identification of potential epitope-based peptide vaccine candidates and significantly downsizes the number of epitopes to be synthesized and analyzed for experimental assays [39]. Therefore, using computer-aided approaches to predict this new kind of vaccine could be a magnificent additive in the way forward of preventing *Cryptococcus neoformans*. Accordingly, this is the first computational-based study to utilize heat shock 70 kDa protein as an attractive immune-proteomic factor that is able to stimulate desirable immune responses against cryptococcosis.

## 2. Materials and Methods

The flow chart demonstrates the overall process of peptide vaccine designing as illustrated in Supplementary Figure 1.

### 2.1. Protein Sequence Retrieval

A total of 38 heat shock 70 kDa protein of *Cryptococcus neoformans* var. *grubii* sequences with a length of 773 mer were retrieved in FASTA format from the National Centre for Biotechnology Information (NCBI) protein database (Accession No. XP_012053205.1) on 30th July 2018 at https://www.ncbi.nlm.nih.gov/protein (Supplementary Table 5).

### 2.2. Determination of the Conserved Regions

The retrieved sequences were aligned to allocate the conserved regions using multiple sequence alignment (MSA). The retrieved antigen sequences were run against the NCBI Reference Protein (RefSeq) using ClustalW as implemented in BioEdit sequence alignment editor software version 7.2.5 [40].

### 2.3. B-Cell Epitope Prediction

B-cell epitopes are determinants on the surface of pathogens that interact with B-cell receptors. B-cell epitopes can be either continuous or discontinuous. Approximately 10% of B-cell epitopes are continuous, consisting of a linear stretch of amino acid. In consequence, the majority of B-cell epitopes are a discontinuous or conformational structure [41, 42].

#### 2.3.1. Continuous B-Cell Epitope Prediction

Analysis of epitope binding affinity to B-cell was assessed by the IEDB B-cell epitope prediction tool at http://tools.iedb.org/bcell/. The classical propensity scale methods and hidden Markov model programmed analysis resource were applied from IEDB to fulfill the following physiochemical criteria (linearity, surface accessibility, and immunogenicity).

*(1) Prediction of Linear B-Cell Epitopes*. BepiPred was conducted as a linear B-cell epitope prediction method to sort out the linear conserved regions with a default threshold value of 0.249 [43].

*(2) Prediction of Surface Accessibility*. The Emini surface accessibility test was implemented to discriminate the surface conserved epitopes with a default cut-off value of 1.000 [44].

*(3) Prediction of Antigenicity*. The Kolaskar and Tongaonkar antigenicity method was used to differentiate the immunogenic sites with a default cut-off value of 1.024 [45].

*(4) Prediction of Beta-Turn*. The Chou and Fasman beta-turn prediction tool was used to predict beta-turn sites with a default cut-off value of 0.950 [46].

*(5) Prediction of Hydrophilicity*. The Parker hydrophilicity prediction tool was utilized to distinguish hydrophilic residues with a default cut-off value of 1.949 [47].

#### 2.3.2. Discontinuous B-Cell Epitope Prediction

The reference sequence of heat shock 70 kDa protein was subjected to the Swiss model in order to get the 3D structure [48]. Based on the geometrical properties of the protein structure, the discontinuous B-cell epitopes were filtered out at the ElliPro prediction tool after submission of the modeled 3D structure at http://tools.iedb.org/ellipro/. ElliPro implements three algorithms evaluating the approximation of the protein shape as an ellipsoid, calculation of residue protrusion index (PI), and clustering of neighboring residues based on their PI values. The minimum score and maximum distance (angstrom) were calibrated in the default mode with a score of 0.5 and 6, respectively [49].

### 2.4. Prediction of T-Cell Epitopes

T-cells identify antigens as a short peptide segment in association with MHC molecules on antigen-presenting cells. There are two categories of T-cells:

1. CD8+ T cytotoxic cells, which recognize peptides displayed by MHC-I molecules
2. CD4+ T helper cells, which recognize epitopes in association with MHC-II molecules

T-cell epitopes only recognize linear peptides. MHC-I binding predictions are now very strong and have wide allelic coverage by integration with predictions of proteasomal cleavage and TAP binding sites [50].

#### 2.4.1. Prediction of MHC-I Binding Profile for Conserved Epitopes

The analysis of epitopes binding to MHC-I molecules was assessed by the IEDB MHC-I prediction tool (version 2013-02-22) at http://tools.iedb.org/mhci/. The functional cleft of MHC-I molecules is closed and can only accommodate short peptides ranging from 9 to 11 amino acids; all epitope lengths were set to the optimum length of 9 mers [51, 52]. Artificial Neural Network (ANN) version 2.2 was chosen as the prediction method as it depends on the median inhibitory concentration (IC_50_) with a default threshold value of 500 nM [53]. It is given that the absolute binding affinity threshold correlates better with immunogenicity [54]. Therefore, a lower IC_50_ value indicates greater binding affinity and vice versa. Based on a rough protocol, all conserved epitopes with an IC_50_ score of less than 50 nM have high affinity, less than 500 nM intermediate affinity, and less than 5000 nM low affinity [55]. Conserved promiscuous epitopes at a score equal to or less than 500 IC_50_ are selected for further analysis whereas epitopes with IC_50_ greater than 500 were omitted.

#### 2.4.2. Prediction of MHC-II Binding Profile for Conserved Epitopes

The analysis of MHC-II selected candidates were assessed by the IEDB MHC-II prediction tool at http://tools.iedb.org/mhcii/. Unlike MHC-I, MHC-II has a flexible pocket with a series of polymorphic pockets and plateaus that interact with several side chains of the peptide core sequence; this provides the specificity of the MHC-peptide interaction and can accommodate peptides of varying lengths, typically 12 to 26 mer. The consensus sequence of the peptides is set to be 9 mer [56]. For the MHC-II binding affinity profile, the most frequent human allele reference set was used. NN-algin was chosen as the prediction method as it depends on the median inhibitory concentration (IC_50_) with a default threshold value of 100 nM [57]. Finally, all conserved immunodominant peptides at a score equal to or less than 100 IC_50_ were selected for further mapping analysis whereas epitopes with IC_50_ greater than 100 were dismissed [58].

### 2.5. Population Coverage Analysis

To ensure the universal coverage within heterogeneous populations, it is crucial to calculate global population coverage for the chosen epitopes since the HLAs are among the most polymorphic proteins and vary among different geographical regions around the world [59] and because the epitopes have a different binding profile with different HLA alleles. Thus, population coverage must be taken into a different set of alleles to cover all regions as possible and to get a desirable immune response in all individuals within a given population. For that reason, all promising MHC-I and MHC-II epitope candidates were assessed for population coverage against the whole world population. The promising candidates were run against different MHC coverage approaches: class I separate, class II separate, and class I and class II combined, through the IEDB population coverage calculation tool at http://tools.iedb.org/population/ [38, 60].

### 2.6. The Physicochemical Properties

The main purpose of vaccination is to induce an immune response after injecting the vaccine into the body. Therefore, it is essential to define the physiochemical parameters associated with the vaccine. The physicochemical properties of vaccine construct were analyzed using BioEdit sequence alignment editor software version 7.2.5 [40] and ExPASy server (ProtParam) [61].

### 2.7. Homology Modeling

The reference sequence of heat shock 70 kDa protein was submitted to Raptor X template-based tertiary structure prediction in order to get the 3D structure [62]. After which, the proposed 3D structure was processed with UCSF chimera 1.13.1 software to visualize and allocate the exact sequential location of the selected promiscuous T-cell and B-cell epitope within heat shock 70 kDa protein [63].

### 2.8. Molecular Docking Analysis

Molecular docking was performed using AutoDock Vina software [64] to predict the strength of binding and binding mode between the two interactive molecules. The 3D structures of the promiscuous epitopes were predicted by PEP-FOLD 3 [65, 66]. The crystal structure of HLA-C∗12:03 (PDB ID 1efx) and HLA-DRB1∗01:01 (PDB ID 2fse) was chosen as a model for molecular docking and was downloaded in a PDB format from the RCSB PDB resource. The selected crystal structures were in a complex form with ligands. Thus, to simplify and to define the potential binding site in the complex structure, all water molecules and ligands were removed by Discovery Studio Visualizer [67]. The partial charge and energy minimization were applied for ligands and targets. Finally, ten independent docking runs were carried out for each peptide. The results were retrieved as binding energies, and the best poses for each epitope that displayed the lowest binding energies with the best intermolecular interaction were visualized using UCSF chimera 1.13.1 software [63], and the 2D interaction was visualized using Discovery Studio Visualizer [67].

## 3. Results

### 3.1. B-Cell Epitope Prediction

**Figure 1:**
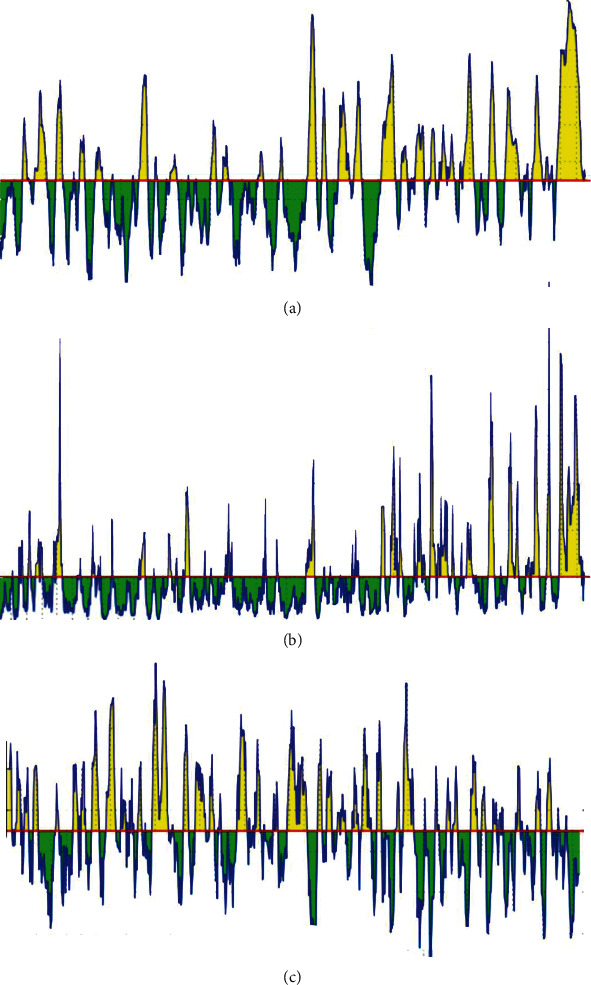
Illustrates the spectrums of the linear conserved surface immunogenic B-cell epitopes. (a) BepiPred Linear Epitope Prediction; the yellow spectrums above and at a cut-off of 0.249 (red line) represent the linear epitopes while the green spectrums exemplify the nonlinear epitopes. (b) Emini surface accessibility prediction; the yellow spectrums above and at a cut-off of 1.000 (red line) illustrate epitopes on the surface whereas green spectrums represent epitopes that are not on the surface. (c) Kolaskar and Tongaonkar antigenicity prediction; the yellow spectrums above and at a cut-off of 1.024 (red line) represent the immunogenic epitopes while green spectrums demonstrate the nonimmunogenic or zerofold epitopes.

**Figure 2:**
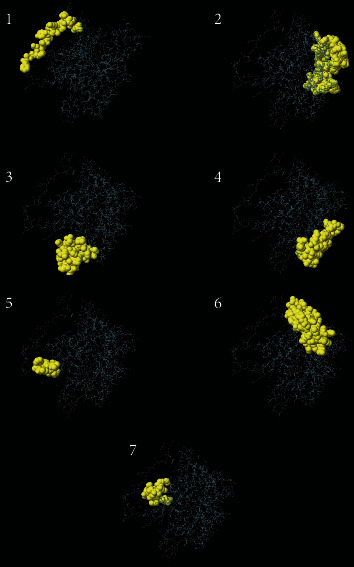
Illustrates the three-dimensional representation of the highest immunogenic discontinuous epitopes (1–7) using the ElliPro prediction tool. The epitopes are depicted in the yellow surface, and the bulk of the heat shock 70 kDa protein is depicted in grey sticks.

In terms of continuous B-cell epitope prediction analysis, the default threshold score of heat shock 70 kDa protein to B-cell was given to be 0.249 in BepiPred Linear Epitope Prediction, and only 298 linear epitopes were predicted. In Emini surface accessibility prediction, the default threshold score of the surface accessibility test was found to be 1.000; 211 epitopes were potentially at the surface by passing the default threshold. In Kolaskar and Tongaonkar antigenicity prediction, the default threshold score of antigenicity was set to be 1.024, fifteen immunogenic epitopes passed the test, and out of all, only eight have passed beta-turn and hydrophilicity prediction tools. Hence, fifteen linear conserved surface antigenic epitopes were passed (Table 1, Figure 1, and Supplementary Figures 11 and 12). However, for the restricted residue length (1-7 mer) implemented in the flexibility prediction tool, testing the flexibility of B-cell epitopes is not feasible. Collectively, four epitopes out of all were predicted to be the promising B-cell epitopes that are able to evoke B lymphocyte for their proper physiochemical properties and length (ANYVQASEK, QSEKPKNVNPVI, SEKPKNVNPVI, and EKPKNVNPVI). With regard to discontinuous B-cell epitope prediction yield, seven promising discontinuous epitopes (Table 2 and Figure 2) were defined from the modeled structure. The predicted epitopes were found to be located on the surface of the protein indicating quick recognition by the host immune system.

### 3.2. Prediction of MHC-I Binding Profile for Conserved Epitopes

We found that 213 conserved epitopes interacted with different MHC-1 alleles. Among the core epitopes, YVYDTRGKL was noticed to be the dominant binder as judged by its interaction with 9 alleles (HLA-A∗02:06, HLA-A∗68:02, HLA-B∗07:02, HLA-C∗03:03, HLA-C∗06:02, HLA-C∗07:01, HLA-C∗12:03, HLA-C∗14:02, and HLA-C∗15:02), followed by LTFYRQGAF, RATPSLVSF, and FTQLVAAYL that interact with 7 alleles for each (Table 3 and Supplementary Tables 1 and 3).

### 3.3. Prediction of MHC-II Binding Profile for Conserved Epitopes

We found that a total of 156 conserved predicted epitopes interacted with a variety of MHC-II alleles. Of those, the core epitope FDYALVQHF was found to be the top binder since it interacts with eleven alleles (HLA-DPA1∗01:03, HLA-DPB1∗02:01, HLA-DPA1∗02:01, HLA-DPB1∗01:01, HLA-DRB1∗01:01, HLA-DRB1∗03:01, HLA-DRB1∗04:05, HLA-DRB1∗07:01, HLA-DRB1∗09:01, HLA-DRB1∗11:01, and HLA-DRB5∗01:01). It is followed by FYRQGAFEL and FFGGKVLNF which are believed to bind with 10 alleles for each (Table 4 and Supplementary Tables 2 and 3).

### 3.4. Physiochemical Parameters

The protein length was found to be 773 amino acids. MW and pI parameters were calculated as 85.69 kDa and 5.12, respectively. The pI value indicates that the protein is acidic in nature. The total numbers of negatively and positively charged residues were 124 and 98, correspondingly. The extinction coefficient of vaccine at 280 nm was measured 67520 M^−1^ cm^−1^ in water. The half-life of the vaccine was predicted to be 30 hours in mammalian reticulocytes (in vitro), >20 hours in yeast (*in vivo*), and >10 hours in *Escherichia coli* (*in vivo*). The instability index was computed to be 35.99, which indicates the thermostability. The aliphatic index and the GRAVY value of the vaccine were determined 85.80 and -0.416, respectively. The high aliphatic index shows that the vaccine is stable in a wide range of temperatures, and the negative GRAVY value indicates vaccine hydrophilicity and has better interaction with the surrounding water molecules. The amino acid composition is shown in Supplementary Figure 8 and Supplementary Table 4.

### 3.5. Population Coverage Analysis

The population coverage test was performed to compute the world coverage of epitopes that bind to separate MHC-I alleles, MHC-II alleles for each, and combined MHC-I and MHC-II and to sort out the most predominant promising epitopes for each coverage mode through the IEDB population coverage analysis tool.

#### 3.5.1. Population Coverage for Isolated MHC-I and MHC-II

**Figure 3:**
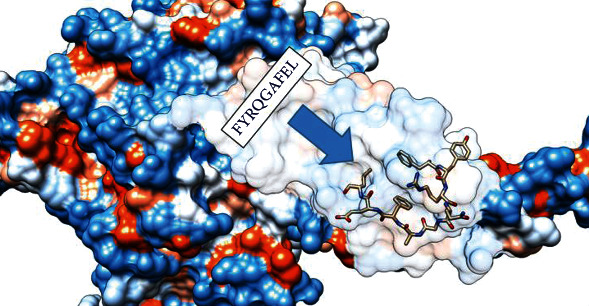
Illustrates the sequential location of FYRQGAFEL as a promising MHC-I & MHC-II epitope, with massive population coverage, within the 3D structure of heat shock 70 kDa protein using UCSF chimera 1.13.1 software.

**Figure 4:**
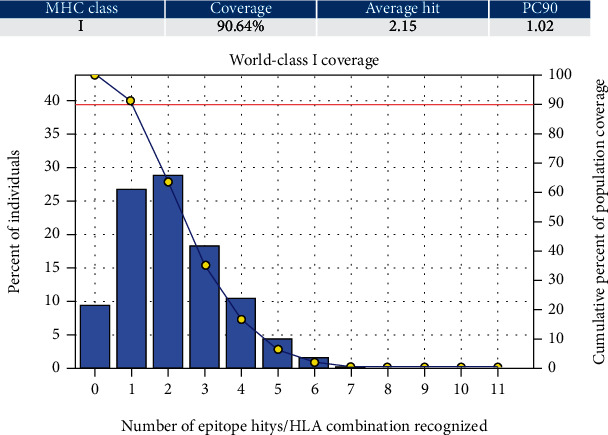
Illustrates the global resident's total percentage for the top three MHC-I epitopes (YVYDTRGKL, FYRQGAFEL, and FTQLVAAYL). Notes: in the graphs, the line (-o) represents the cumulative percentage of population coverage of the epitopes; the bars represent the population coverage for each epitope.

Three epitopes had exhibited the highest coverage percentage (YVYDTRGKL, FYRQGAFEL, and FTQLVAAYL) in the isolated MHC-I mode. The maximum population coverage percentage over these epitopes was found to be 60.93% for YVYDTRGKL (Table 5). In the case of MHC class II (Table 5), three epitopes had exhibited the best coverage percentage (FFGGKVLNF, FYRQGAFEL, and FDYALVQHF). The highest coverage percentage of these epitopes was awarded to FFGGKVLNF with a percentage of 98.02%. For the top three coverage epitopes together in MHC-I and MHC-II, for each was found to be 90.64% and 99.3%, respectively (Table 5 and Figures 3 and 4).

#### 3.5.2. Population Coverage for Combined MHC-I and MHC-II

**Figure 5:**
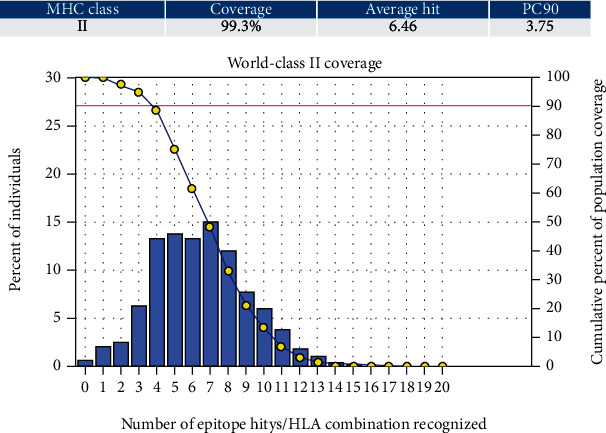
Illustrates the global population for the top three MHC-II epitopes (FFGGKVLNF, FYRQGAFEL, and FDYALVQHF). Notes: in the graph, the line (-o) represents the cumulative percentage of population coverage of the epitopes; the bars represent the population coverage for each epitope.

Three epitopes had exhibited the highest coverage percentage (FFGGKVLNF, FYRQGAFEL, and FINAQLVDV). The most abundant coverage percentage of these epitopes in the world was granted to FFGGKVLNF with a percentage of 98.20%. For the top three coverage epitopes, the coverage percentage together was found to be 99.77% (Table 5, Figure 5, and Supplementary Table 3).

### 3.6. Homology Modeling

**Figure 6:**
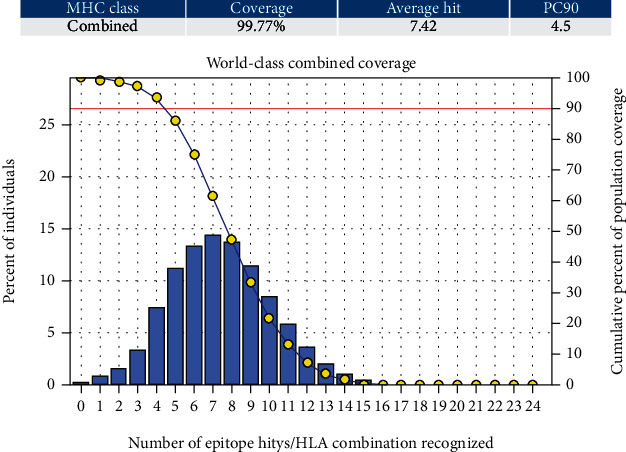
Illustrates the global population for the top three MHC-I & II epitopes in combined mode (FFGGKVLNF, FYRQGAFEL, and FINAQLVDV). Notes: in the graphs, the line (-o-) represents the cumulative percentage of population coverage of the epitopes; the bars represent the population coverage for each epitope.

The 3D structure of the heat shock 70 kDa protein complex of *Cryptococcus neoformans* var. *grubii* and the sequential location of FYRQGAFEL were a promising MHC-I and MHC-II epitope, with massive population coverage, within the 3D structure of heat shock 70 kDa protein (Figure 6 and Supplementary Figures 2–7).

### 3.7. Molecular Docking Analysis

The molecular docking result of the promiscuous epitopes that showed the best binding affinity in terms of their binding energies and modes is shown in Table 6, Figures 7–12, and Supplementary Figures 9 and 10.

## 4. Discussion

The overall analysis revealed seventeen promiscuous B-cell and T-cell epitopes that consist of four immunogenic continuous B-cell epitopes (ANYVQASEK, QSEKPKNVNPVI, SEKPKNVNPVI, and EKPKNVNPVI), seven discontinuous B-cell epitopes, and six immunogenic MHC-I and MHC-II epitopes (YVYDTRGKL, FYRQGAFEL, FTQLVAAYL, FFGGKVLNF, FINAQLVDV, and FDYALVQHF) that are proposed to be used in epitope-based vaccine designing.

The importance of Hsp70 family proteins as stand-alone immune response modulators is a widely held view since it prolongs the survival rate of the animal model by decreasing *C. neoformans* cell burden from the CNS of rabbits and pulmonary fungal growth clearance in infected mice [21–23, 68]. Several lines of evidence have suggested many of the heat shock protein family as potential candidates in designing a recombinant vaccine in mouse models; Hsp90 in *Candida*, Hsp60 in *Histoplasma*, and Hsp70 in *Schistosoma* [27–29]. Marañón and coworkers [69] conducted an *in silico* and *in vivo* integrative approach on Hsp70 of *Trypanosoma cruzi* and found that four immunodominant epitopes (TLLTIDGGI, DSLTNLRAL, TLQPVERVL, and RIPKVMQLV) were assayed for their recognition by CTL of HLA-A∗02:01 and *T. cruzi*-infected transgenic B6-A2/Kb mice. Of those, TLQPVERVL and RIPKVMQLV were also recognized by CTL of HLA-A∗02:01 Chagas disease patients, indicating that these peptides are processed and displayed as MHC-I epitopes during the natural history of *T. cruzi* infection. Khalil's group [70] demonstrated the immunoreactive mannoprotein (MP88) of *Cryptococcus neoformans* var. *grubii* using an immunoinformatic approach and found three potential MHC-I and MHC-II epitopes for each (YMAADQFCL, VSYEEWMNY, and FQQRYTGTF) and (YARLLSLNA, ISYGTAMAV, and INQTSYARL) correspondingly and four promising B-cell epitopes (AYSTPA, AYSTPAS, PASSNCK, and DSAYPP). Nooney et al. [71] reported a recombinant antibody, namely, mycograb, which targets an epitope within the Hsp90 of *C. albicans* which is conserved with the corresponding protein in *C. neoformans*. The study concluded that mycograb and amphotericin B can act in a synergistic fashion against multiple *Candida* species and *C. neoformans* clinical isolates. Khan and coworkers [72] identified that fibrin microsphere-based targeted delivery of cytosolic proteins is able to induce robust protective immune responses against experimental murine cryptococcosis. Together, these studies support our findings and point toward the fact that the development of a cryptococcal vaccine is feasible and possible through screening the *Cryptococcus neoformans*' immunogenic proteins and utilizing the promising antigenic epitopes in peptide vaccine designing. A therapeutic vaccine is able to prevent reactivation and is effective in the setting of established cryptococcosis [32, 73, 74].

An overview of a cryptococcal vaccine has already been provided in a perspective review article by Ueno and coworkers [75], extensively discussing many aspects in “Vaccines and Protective Immune Memory against Cryptococcosis.” There are many experiments with conventional vaccines in the *C. neoformans* field. For example, killed vaccines have generally been ineffective and some have enhanced infection. Live vaccines using attenuated mutants have been shown to induce stronger, longer-lasting immune responses in those immunocompetent [76–78]. However, live vaccines are not safe for use in immunocompromised patients, and any attempt to develop a live vaccine for cryptococcosis is likely to face significant ethical outcomes. In contrast, the success of subunit and conjugate vaccines against hepatitis B virus, *Haemophilus influenza* type B, and *Streptococcus pneumonia* has shown the safety and effectiveness of this approach [79]. Several theories have utilized the component of a cryptococcal capsule as a GXM-based vaccine; immunization by a GXM-based vaccine even conjugated with a protein or tetanus toxoid (GXM-TT) failed to induce a specific protective immune response and often acts as a deleterious factor [32, 73]. Upadhya and coworkers [80] found that heat-killed chitosan of cryptococcal cell wall vaccines could develop robust protective immunity against virulent strains of *C. neoformans* in mice which represent a potential vaccine candidate. Patients with suppressed T-cell responses will undoubtedly suffer from reduced memory responses and disease relapses, rendering conventional vaccine strategies useless. Hence, implementing novel combined T-cell and B-cell vaccines that have the potential to mediate protective immunity against *C. neoformans* would improve the quality of life of immunocompromised patients [81] and provide a rationale to support continued investment in Cryptococcus vaccine research [74]. Nonetheless, the efficacy of the Cryptococcus vaccine candidate to induce protection against cryptococcosis will need to be confirmed using an immune-deficient animal model system to mimic immune suppression in human populations.

Molecular docking and population coverage analysis are crucial factors in the development and refinement of epitope selection. The epitope FYRQGAFEL had shown an exceptional result in terms of its broad spectrum of binding with MHC-I, MHC-II, and population coverage percentage; alongside, it displayed the strongest binding affinity, when docked with HLA-C∗12:03 over the top promising MHC-I epitopes. Despite this outstanding coverage and binding scores, the aforementioned epitope was not considered one of the abundant binders to MHC-I, a possible explanation for its binding to one or more of the commonly occurring MHC-I alleles among global residents. The epitope FDYALVQHF also showed the highest negative free energy of binding with HLA-DRB1∗01:01 over the top promising MHC-II epitopes, revealing a stronger interaction between the epitope and HLA. Besides, it had the most abundant binding profile to MHC-II alleles and had the top population coverage. The core epitope FFGGKVLNF had the most dominant population coverage in MHC-II and combined mode but showed the weakest binding affinity to HLA-DRB1∗01:01 among the promising MHC-II epitopes. Regarding MHC-I binding and population coverage, our finding has shown that YVYDTRGKL had the utmost binding profile and coverage with the highest global energy among the promising MHC-I epitopes. Apart from the Δ*G* binding value, the interaction between epitope and HLA can also be studied by analyzing the intermolecular interaction between them. The 2D interaction analysis revealed that more hydrogen bonds were present in FYRQGAFEL–HLA-C∗12:03 than in FYRQGAFEL–HLA-DRB1∗01:01 complexes. However, the Δ*G* binding value for FYRQGAFEL–HLA-C∗12:03 has shown a more negative value. This is maybe due to the inequality of hydrogen bonds and the contribution of other intermolecular interactions, which depend on the atom distances and angles (Supplementary Figures 11 and 12).

A limited number of validated sequences were retrieved due to the lack of equivalent data in the literature; biases could be incorporated. Furthermore, regarding HLA allele frequencies and reference sets with population coverage, there is no predictor for HLA-DRB5∗01:01, HLA-DPA1∗01, and HLA-DRB3∗01:01 at the IEDB population coverage tool, which might mislead the inference of coverage percentage. However, there is a definite need for experimental validation for the carefully chosen vaccine candidates *in vitro* and *in vivo* to fortify their antigenic and immunogenic potentials using a high-density peptide array. Additionally, further computational studies are needed to be conducted in the pathogen-derived heat shock protein family, as it is believed to find universal epitopes that could be utilized as a peptide vaccine against other pathogen-derived Hsp. Finally, *C. neoformans* expresses a significant number of immune-proteomic factors that could help the parasite to evade and evoke host immunity. Thus, screening of new immune-proteomic factors may facilitate the future development of immunotherapeutic interventions aimed at boosting human being immunity against cryptococcosis.

Theoretically, no single epitope vaccine would provide universal protection against all *Cryptococcus neoformans* var. *grubii* strains because of allelic polymorphism among the global population, and epitopes might have a different binding profile with different HLA alleles [38, 59]. Nevertheless, complete protection could be achieved by combining multiple epitopes through targeting immunodominant regions comprising of multiple epitopes. Our prime vaccine candidate was a putative ten antigenic continuous B-cell and T-cell epitope (ANYVQASEK, QSEKPKNVNPVI, SEKPKNVNPVI, EKPKNVNPVI, YVYDTRGKL, FYRQGAFEL, FTQLVAAYL, FFGGKVLNF, FINAQLVDV, and FDYALVQHF) and seven discontinuous B-cell epitopes. Together, these epitopes are forecasted to trigger T lymphocytes, B lymphocytes, and immunological memory with overall coverage above 90%. Accordingly, this *in silico* vaccine is expected to be the future epitope-based peptide vaccine with potential immunogenicity that is able to stimulate desirable immune responses against all strains of *Cryptococcus neoformans* var. *grubii* with massive global population coverage.

## 5. Conclusion

Cryptococcosis is a serious global problem concerning morbidity and mortality in immunocompromised individuals. Unfortunately, the unavailability of vaccines and the failure of antifungals against cryptococcosis have led to affecting many precious lives in various regions of the world. This *in silico* analysis provides novel insights regarding computational vaccinology that aids in the design and discovery of novel vaccine candidates. This study proposed promising epitopes (ANYVQASEK, QSEKPKNVNPVI, SEKPKNVNPVI, EKPKNVNPVI, YVYDTRGKL, FYRQGAFEL, FTQLVAAYL, FFGGKVLNF, FINAQLVDV, and FDYALVQHF) that might possess the therapeutic and prophylactic potentials to combat lethal cryptococcosis in immunocompromised patients. Accordingly, this *in silico* vaccine is expected to be the future epitope-based peptide vaccine with potential immunogenicity that is able to stimulate desirable immune responses against all strains of *Cryptococcus neoformans var. grubii* with massive global population coverage. Therefore, we recommend assessing its *in vitro* and *in vivo* antigenic potential through experimental validation.

## Figures and Tables

**Figure 7:**
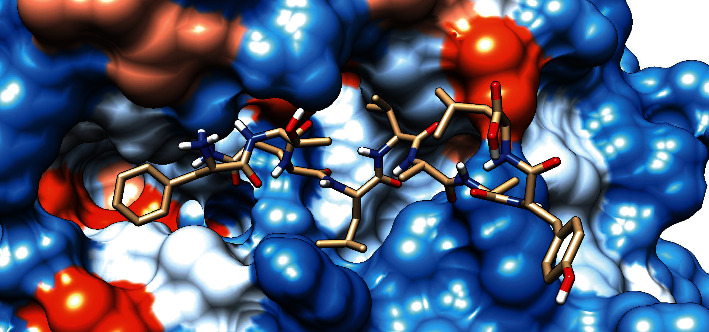
Illustrates the 3D interaction of the best docking poses of FTQLVAAYL in the binding sites of HLA-C∗12:03.

**Figure 8:**
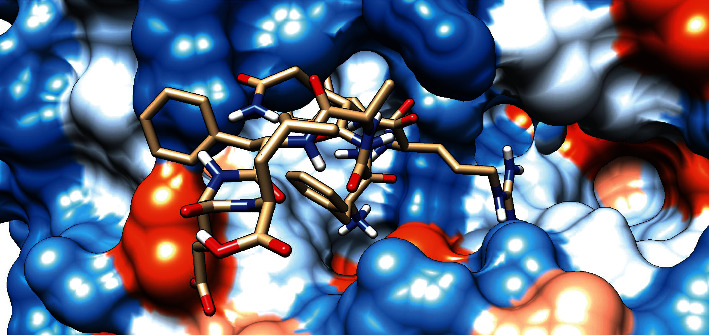
Illustrates the 3D interaction of the best docking poses of FYRQGAFEL in the binding sites of HLA-C∗12:03.

**Figure 9:**
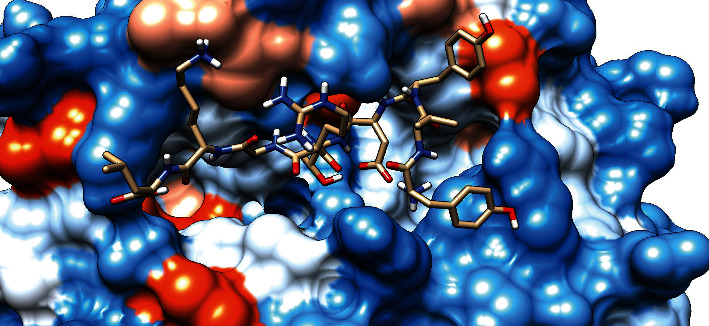
Illustrates the 3D interaction of the best docking poses YVYDTRGKL in the binding sites of HLA-C∗12:03.

**Figure 10:**
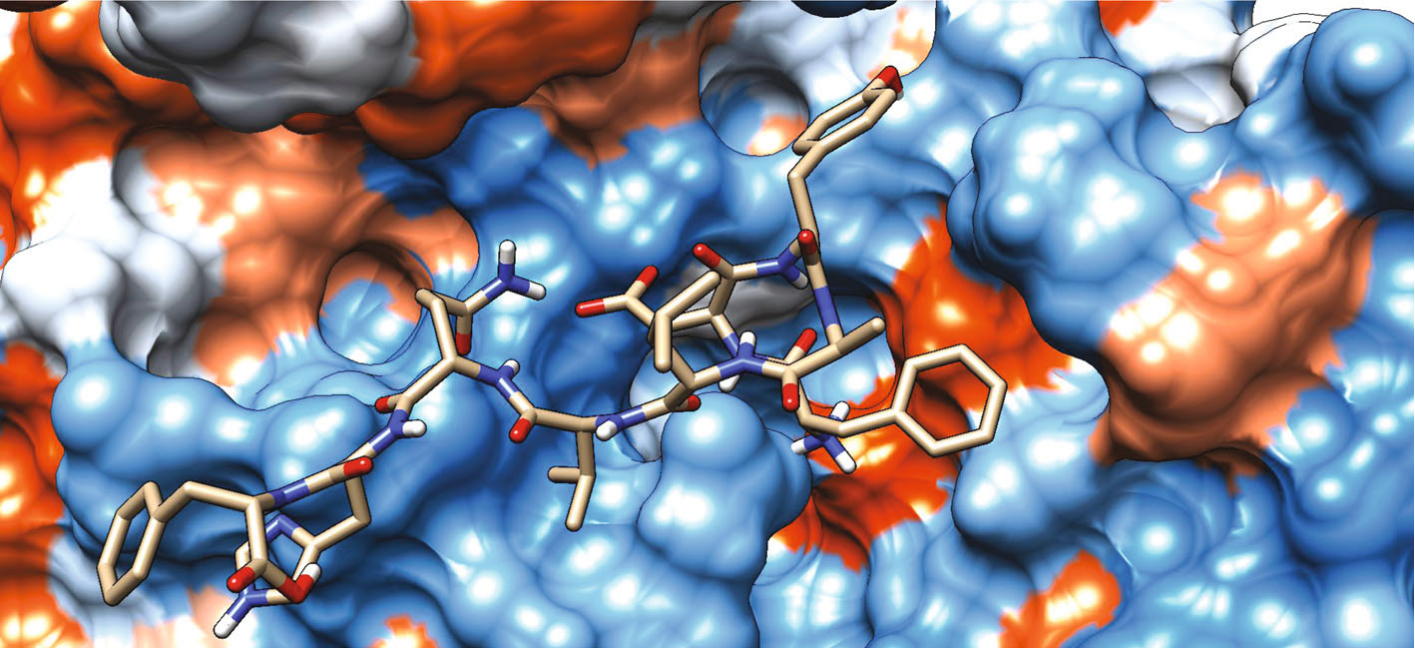
Illustrates the 3D interaction of the best docking poses FDYALVQHF in the binding sites of HLA-DRB1∗01:01.

**Figure 11:**
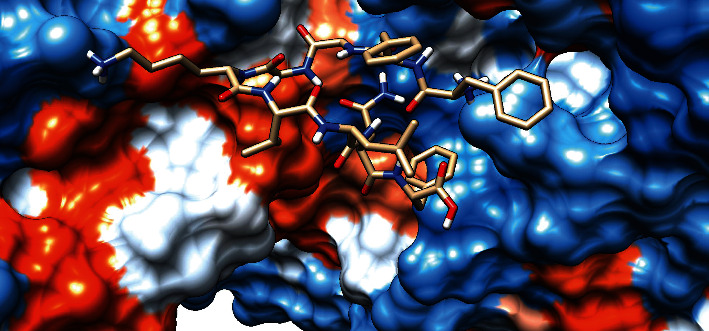
Illustrates the 3D interaction of the best docking poses FFGGKVLNF in the binding sites of HLA-DRB1∗01:01.

**Figure 12:**
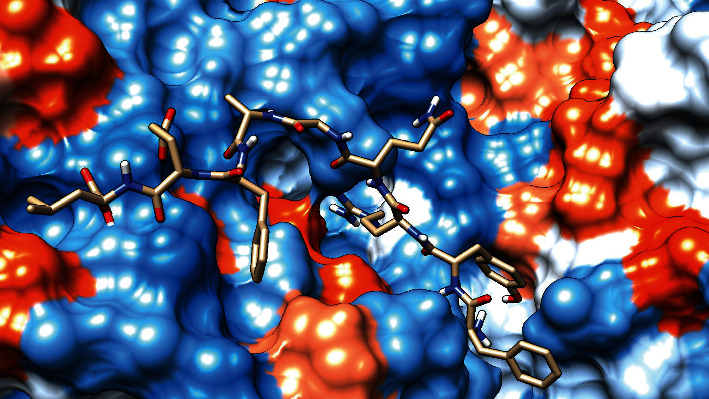
Illustrates the 3D interaction of the best docking poses FYRQGAFEL in the binding sites of HLA-DRB1∗01:01.

**Table 1:** List of the fifteen linear conserved surface antigenic epitopes of heat shock 70 kDa protein with their surface accessibility score, antigenicity score, beta-turn score, and hydrophilicity score.

Start	End	Linear peptide^a^	Length	Surface score^b^	Antigenicity score^c^	Beta-turn score^d^	Hydrophilicity score^e^
79	84	PEVEEY	6	1.748	1.027	0.897	3.317
592	600	ANYVQASEK^∗^	9	1.243	1.028	0.964	3.511
593	600	NYVQASEK	8	1.625	1.024	1.002	3.688
594	600	YVQASEK	7	1.335	1.059	0.923	3.214
595	600	VQASEK	6	1.128	1.042	0.887	4.067
468	474	VKSVEKP	7	1.118	1.079	0.959	2.914
705	716	QSEKPKNVNPVI^∗^	12	1.3	1.028	1.067	2.875
706	716	SEKPKNVNPVI^∗^	11	1.002	1.029	1.075	2.591
707	716	EKPKNVNPVI^∗^	10	1.003	1.031	1.039	2.2
504	513	EVEKEEEVTV	10	1.115	1.024	0.717	3.88
735	741	ILNKPKP	7	1.257	1.024	1.097	0.771
735	745	ILNKPKPKPKV	11	1.844	1.043	1.065	1.382
735	746	ILNKPKPKPKVT	12	1.995	1.032	1.057	1.7
469	476	KSVEKPAS	8	1.544	1.031	1.037	4.088
749	756	TPQQPPAQ	8	2.997	1.026	1.14	3.95

^∗^Top promising epitopes for their ideal length and physiochemical properties. ^a^BepiPred Linear default threshold value 0.249. ^b^Emini surface accessibility default threshold value 1.000. ^c^Kolaskar and Tongaonkar antigenicity default threshold value 1.024. ^d^Chou and Fasman beta-turn default threshold value 0.950. ^e^Parker hydrophilicity default threshold value 1.949.

**Table 2:** List of predicted discontinuous B-cell epitopes of heat shock 70 kDa protein by ElliPro prediction tool with the number of residues and their scores.

No.	Residues	No. of residues	Score
1	A:Q46, A:F393, A:A394, A:V395, A:H396, A:D397, A:I398, A:A399, A:A400, A:Y401, A:P402, A:I403, A:K404, A:I405, A:S406, A:W407, A:E408	17	0.912
2	A:Q243, A:H244, A:F245, A:A246, A:E247, A:E248, A:F249, A:K250, A:T251, A:K252, A:Y253, A:I255, A:D256, A:V257, A:L258, A:S259, A:S260, A:P261, A:K262, A:A263, A:R266, A:V277, A:L278, A:A280, A:N281, A:T282, A:E283, A:A284, A:P285, A:I286, A:N287, A:V288, A:E289, A:S290, A:L291, A:M292, A:N293, A:D294, A:I295, A:D296, A:A297, A:T298, A:S299, A:T300, A:L301, A:T302, A:R303, A:E304, A:S305, A:E307, A:K308	51	0.777
3	A:G183, A:I184, A:T185, A:K186, A:A187, A:D188, A:L189, A:P190, A:E191, A:S192, A:T193, A:E194, A:A195, A:P196, A:R197, A:H198, A:A215, A:F216, A:S217, A:K218, A:G219, A:Q220, A:T222, A:I335, A:D336, A:A337	26	0.729
4	A:K224, A:T310, A:D311, A:H312, A:S315, A:R316, A:S318, A:V319, A:A322, A:E323, A:A324, A:L325, A:E326, A:K327, A:A328, A:G329, A:L330, A:T331, A:I332, A:D333, A:Q334, A:E352, A:R353, A:Q355, A:Q356, A:F357, A:F358, A:G359, A:G360, A:K361, A:V362, A:L363	32	0.69
5	A:A19, A:R20, A:H21, A:G23, A:A382, A:S383, A:L384, A:S385, A:P386, A:V387, A:F388	11	0.665
6	A:T43, A:P44, A:R45, A:A56, A:S59, A:N60, A:F61, A:K62, A:N63, A:T64, A:L71, A:R74, A:S75, A:F76, A:N77, A:D78, A:P79, A:E80, A:V81, A:E82, A:E83, A:K86, A:K87, A:F88, A:N90, A:A91, A:Q92, A:L93, A:V94, A:D95, A:V96, A:N97, A:G98, A:E99, A:I100, A:G101, A:K103, A:V104, A:N105, A:Y106, A:L107, A:G108, A:E109, A:P110, A:T111, A:D112, A:F113	47	0.655
7	A:V4, A:I27, A:A131, A:A132, A:E133, A:L134, A:K135, A:Q136, A:S137, A:V138, A:S139, A:D140, A:A164, A:G165, A:L166, A:N167	16	0.537

**Table 3:** List of promising epitopes of heat shock 70 kDa protein that had the highest binding affinity with MHC-I alleles in terms of IC_50_ and percentile rank.

Epitopes	Start	End	Allele	IC_50_	Percentile
YVYDTRGKL^∗^	579	587	HLA-A∗02:06	274.73	1.8
	579	587	HLA-A∗68:02	359.64	1.5
	579	587	HLA-B∗07:02	488.64	1.3
	579	587	HLA-C∗03:03	10.15	0.06
	579	587	HLA-C∗06:02	350.13	0.13
	579	587	HLA-C∗07:01	133.84	0.04
	579	587	HLA-C∗12:03	10.95	0.03
	579	587	HLA-C∗14:02	10.45	0.02
	579	587	HLA-C∗15:02	475.11	0.24
FTQLVAAYL^∗^	115	123	HLA-A∗02:01	387.43	2.6
	115	123	HLA-A∗02:06	166.27	1.4
	115	123	HLA-A∗68:02	21.89	0.22
	115	123	HLA-C∗03:03	335.75	0.6
	115	123	HLA-C∗05:01	356.66	0.18
	115	123	HLA-C∗14:02	74.62	0.14
	115	123	HLA-C∗15:02	276.33	0.16
FYRQGAFEL^∗^	437	445	HLA-A∗23:01	163.92	0.47
	437	445	HLA-A∗24:02	379.48	0.6
	437	445	HLA-C∗03:03	234.95	0.5
	437	445	HLA-C∗07:02	27.33	0.02
	437	445	HLA-C∗12:03	429.65	0.5
	437	445	HLA-C∗14:02	10.8	0.02

^∗^Top promising epitopes with strong binding affinity and massive global population coverage.

**Table 4:** List of the three promising epitope core sequences of heat shock 70 kDa protein that had the highest binding affinity with MHC-II in terms of IC_50_ and percentile ranks.

Core sequence	Start	End	Allele	Epitope sequence	IC_50_	Rank
FDYALVQHF^∗^	234	248	HLA-DPA1∗01:03	GRDFDYALVQHFAEE	86.1	7.57
	234	248	HLA-DPB1∗02:01	GRDFDYALVQHFAEE	86.1	7.57
	235	249	HLA-DPA1∗02:01	RDFDYALVQHFAEEF	40.4	4.14
	235	249	HLA-/DPB1∗01:01	RDFDYALVQHFAEEF	40.4	4.14
	234	248	HLA-DRB1∗01:01	GRDFDYALVQHFAEE	54.3	22.25
	233	247	HLA-DRB1∗03:01	GGRDFDYALVQHFAE	28.2	1.65
	234	248	HLA-DRB1∗04:05	GRDFDYALVQHFAEE	21.1	1.41
	232	246	HLA-DRB1∗07:01	FGGRDFDYALVQHFA	10.7	1.8
	235	249	HLA-DRB1∗09:01	RDFDYALVQHFAEEF	81.3	5.61
	234	248	HLA-DRB1∗11:01	GRDFDYALVQHFAEE	65.9	10.43
	234	248	HLA-DRB5∗01:01	GRDFDYALVQHFAEE	70.8	12.49
FFGGKVLNF^∗^	353	367	HLA-DPA1∗01:03	RIQQFFGGKVLNFTL	57.8	5.71
	353	367	HLA-DPB1∗02:01	RIQQFFGGKVLNFTL	57.8	5.71
	354	368	HLA-DPA1∗02:01	IQQFFGGKVLNFTLN	43.6	4.53
	354	368	HLA-DPB1∗01:01	IQQFFGGKVLNFTLN	43.6	4.53
	355	369	HLA-DPA1∗03:01	QQFFGGKVLNFTLNQ	72.6	7.73
	355	369	HLA-DPB1∗04:02	QQFFGGKVLNFTLNQ	72.6	7.73
	352	366	HLA-DQA1∗05:01	ERIQQFFGGKVLNFT	14.4	2.46
	352	366	HLA-DQB1∗03:01	ERIQQFFGGKVLNFT	14.4	2.46
	353	367	HLA-DRB1∗01:01	RIQQFFGGKVLNFTL	20.4	11.5
	353	367	HLA-DRB1∗07:01	RIQQFFGGKVLNFTL	29.2	5.56
FYRQGAFEL^∗^	433	447	HLA-DPA1∗01	KVLTFYRQGAFELEA	67.8	3.94
	433	447	HLA-DPB1∗04:01	KVLTFYRQGAFELEA	67.8	3.94
	433	447	HLA-DPA1∗01:03	KVLTFYRQGAFELEA	63.4	6.11
	433	447	HLA-DPB1∗02:01	KVLTFYRQGAFELEA	63.4	6.11
	434	448	HLA-DPA1∗02:01	VLTFYRQGAFELEAA	36.5	3.66
	434	448	HLA-DPB1∗01:01	VLTFYRQGAFELEAA	36.5	3.66
	434	448	HLA-DRB1∗01:01	VLTFYRQGAFELEAA	5.7	1.43
	431	445	HLA-DRB1∗07:01	STKVLTFYRQGAFEL	19.1	3.63
	434	448	HLA-DRB1∗09:01	VLTFYRQGAFELEAA	51.5	3.35

^∗^Top promising epitopes with strong binding affinity and massive global population coverage.

**Table 5:** List of global population coverage for promising epitopes of heat shock 70 kDa protein in isolated MHC class I & II and combined class I&II.

Core peptide	World coverage class I	Total HLA hits	Core peptide	World coverage class II	Total HLA hits	Core peptide	World coverage class I&II combined	Total HLA hits
YVYDTRGKL^∗^	60.93%	9	FFGGKVLNF^∗^	98.02%	10	FFGGKVLNF^∗^	98.20%	12
FYRQGAFEL^∗^	55.50%	6	FYRQGAFEL^∗^	95.39%	10	FYRQGAFEL^∗^	97.95%	16
FTQLVAAYL^∗^	55.41%	7	FDYALVQHF^∗^	95.38%	11	FINAQLVDV^∗^	96.76%	10
FACASLSPV	55.16%	6	VVFGTANPI	77.79%	6	FDYALVQHF	96.32%	14
LLSRVSVPL	53.01%	5	FTQLVAAYL	61.30%	8	LVQHFAEEF	95.27%	8
YADPASLPK	45.71%	6	FKNTVGSLK	36.46%	5	FSFTQLVAA	92.03%	8
RATPSLVSF	38.17%	7	IAGLNALRL	56.08%	7	FACASLSPV	87.11%	11
GIMNFEGAY	36.03%	6	LKRLIGRSF	42.52%	5	FTQLVAAYL	82.74%	15
LTFYRQGAF	35.53%	7	IVKVKARLN	39.32%	5	AAAALREAL	79.45%	6
FVDVGHSDY	34.93%	5	LREALNTYL	36.70%	5	YVYDTRGKL	71.62%	12

^∗^Top promising epitopes with massive population coverage.

**Table 6:** List of the molecular docking result of the promiscuous epitopes that showed the best binding affinity in terms of their binding energies.

Epitope	Binding MHC molecule	Binding energy (^∗^Δ*G* kcal/mol)
FDYALVQHF	HLA-DRB1∗01:01	-19.03
FFGGKVLNF	HLA-DRB1∗01:01	-17.23
FYRQGAFEL	HLA-DRB1∗01:01	-17.61
FTQLVAAYL	HLA-C∗12:03	-15.38
FYRQGAFEL	HLA-C∗12:03	-25.2
YVYDTRGKL	HLA-C∗12:03	-30.40

^∗^Global energy; the energy required to estimate the strength of association between the epitope within the active cleft of MHC molecules; more negative value indicates favored and stable binding of the complex.

## Data Availability

All the analyzed data during this study are included in this manuscript and its supplementary files.
